# Functionalization and antimicrobial evaluation of ampicillin, penicillin and vancomycin with *Pyrenacantha grandiflora* Baill and silver nanoparticles

**DOI:** 10.1038/s41598-020-68290-x

**Published:** 2020-07-14

**Authors:** Arinao Murei, Wasiu B. Ayinde, Mugera W. Gitari, Amidou Samie

**Affiliations:** 1 Molecular Parasitology and Opportunistic Infections Program, Department of Microbiology, School of Mathematical and Natural Sciences, University of Venda, Private Bag X5050, Thohoyandou, 0950 South Africa; 2 Environmental Remediation and Water Pollution Chemistry Group (ERWPCG), Department of Ecology and Resource Management, School of Environmental Sciences, University of Venda, Private Bag X5050, Thohoyandou, Limpopo Province 0950 South Africa

**Keywords:** Environmental biotechnology, Bioanalytical chemistry

## Abstract

Some antibiotics have lost their efficacy over common infections and this has led to the search for new antibiotics and chemically altering existing ones for a better control of infectious diseases. In the present study, *Pyrenacantha grandiflora* tubers extracts were conjugated with ampicillin, penicillin, vancomycin and silver nanoparticles and their antimicrobial activity was evaluated against *Escherichia coli*, *Staphylococcus aureus* and *Klebsiella Pneumoniae*. The reactions were confirmed by formation of new functional groups that were identified by Fourier transmission infrared spectroscopy (FTIR). Minimum inhibitory concentrations were determined using the microdilution assay. Minimum bactericidal concentrations and the fractional inhibition concentration index were also determined. FTIR analysis indicated different functional group associated with conjugation. The activity of ampicillin was improved when conjugated with silver nanoparticles against *K. pneumonia* and *E. coli*. Vancomycin showed improvement of activity when conjugated to silver nanoparticles against *K. pneumonia*. Penicillin was improved by acetone extracts and vancomycin showed to be more effective when conjugated with silver nanoparticles and water extracts. The conjugation of *P. grandiflora* with penicillin, ampicillin and vancomycin in the presence of silver nanoparticles improved their biological activities. Therefore, the conjugates are medicinally important and can be used to improve the activity of existing antibiotics.

## Introduction

The occurrence of infections brought about by various pathogens mainly bacteria have been a challenge faced by the world, and the advancement of antibiotics resistance is threatening our capacity to treat regular ailments. Hence, bringing about protracted diseases, disability, and death^1^. Bacterial resistance is of significant financial importance, and some engineered compound have unwanted side effects. Therefore it is essential and basic to look for new and less expensive molecules with lesser side effects^2^. Multi drug resistance of some strains (*Escherichia coli*, *Staphylococcus aureus*, and *Klebsiella pneumoniae)* cause health care associated infections in hospitals and have evolved drug resistance to many of the established antimicrobial compounds, in the market at present^3^. For example, a mutation in DNA gyrase enzyme results in resistance towards quinolones in *E. coli* and *S. pneumonia*^4^*. Staphylococcus aureus* has developed resistance against vancomycin and mutations affecting RNA polymerase β subunit have resulted in *E. coli* resistance towards rifampicin^5^. Some generally utilized antibiotics have lost their efficacy in curing infection, this includes penicillin and other β-lactam drugs and this add to the dilemma, which requires the quick actions to enhance drug design, discovery, and delivery.

For so many years medicinal plants have been utilized in the treatment of human diseases and many studies show their effectiveness as antimicrobial agents. For many years *Pyrenacantha grandiflora* has been used traditionally in treatment and management of diarrhea, tooth pain and gastrointestinal infections. From a previous study, *P. grandiflora* has been evaluated for antimicrobial activity and was shown to be effective against *Aeromonas* spp^6,7^.

Antibiotics such as penicillin, ampicillin, and vancomycin are rendered obsolete since they have lost their efficacy in curing common illnesses. Scientists are however still trying to enhance the activity of less active antibiotics by co-synthesis of nanoparticles with those antibiotics. These antibiotics have been conjugated to nanoparticles and led to increased antimicrobial activity against multidrug resistant microorganisms including methicillin-susceptible *Staphylococcus aureus* and methicillin-resistant *Staphylococcus aureus*^8^.

In the present study, medicinal plants and silver nanoparticles are emerging to address the challenge due to their efficacy as antimicrobial agents. Over the past years, silver nanoparticles (AgNPs) have shown very great activity on food, cosmetics, and beverage industries^9^. Number of studies have proven that AgNPs have excellent antimicrobial, anti-platelet, anti-inflammatory, anti-angiogenic, anticancer and antiviral activities accompanied by a smaller amount of toxicity and further improve the quality of biodegradability and bioavailability in industrial applications^10,11,12,13^. However, research has been focused on developing several strategies to this issue by making novel antibacterial agents or artificially adjusting the action of current cost effective antibiotics. The discoveries and development of novel compounds from medicinal plants has some advantages such as efficiency and cost effectiveness.

In most studies, an increase in antimicrobial activity was reported after conjugation of antibiotic to nanoparticles. Hence, AgNPs were reported to improve the antimicrobial activity of ampicillin^14^. Silver nanoparticle was also reported to have antibacterial activity when synthesized using plant extract^15^. However, the combination of the three materials (Silver nanoparticles, medicinal plant extracts and antibiotics) has not been done before. This study aimed at preparing and evaluating the antibacterial activity of *Pyrenacantha grandiflora* Baill extracts when conjugated with silver nanoparticles and antibiotics (vancomycin, ampicillin and penicillin) against growth of pathogenic bacteria (*Staphylococcus aureus*, *Escherichia coli*, *Klebsiella pneumoniae*) in order to achieve synergistic effect and thus offer advantages such as dosage compliance, minimizing toxicity and overcoming drug resistance when compared to the parent counterparts^16^.

## Materials and methods

### Used microorganism, growth conditions and antibiotics

The microorganisms that were used in this study included methicillin-resistant *Staphylococcus aureus* (ATCC 25,923), *Beta* lactamase producing *Klebsiella pneumoniae* (ATCC 700,603) and *Beta* lactamase producing *Escherichia coli* (ATCC 35,218). These bacterial strains were maintained on mannitol salt agar, MacConkey agar, and nutrient agar respectively. All media were purchased from Rochelle Chemicals (Johannesburg, South Africa). An inoculum of each bacterial strain was suspended in 5 ml of Mueller Hinton broth (Rochelle chemicals, SA) and incubated for 3 h at 37 °C. The cultures were diluted with Mueller Hinton Broth and adjusted to give a concentration of bacterial cells equivalent to a McFarland 0.5 standard prior to the antibacterial testing. The following antibiotics were used in this study: Penicillin (Sigma, Germany), Vancomycin (Fluka Chemie, Denmark) and Ampicillin (Sigma-Aldrich, USA).

### Silver nanoparticles preparation

Silver nanoparticles were prepared using Turkevich protocol (1951). Briefly, 1 mM of silver nitrate (AgNO_3_) powder (Sigma, USA) and 1% tri-sodium citrate of analytical grade purity were used. The silver colloid was prepared by using chemical reduction method. A total of 100 ml of 1 mM AgNO_3_ was heated to boiling, to which 5 ml of 1% tri-sodium citrate was added drop by drop. During the process, the solution was mixed vigorously. The solution was heated until colour's change is evident (yellowish brown). Then it was removed from the heating plate and stirred until cooled to room temperature.

### UV–Vis spectrum analysis

The characterization of silver nanoparticles was done using UV–Visible Spectrophotometer (Specord 210, Analytikjena spectrometer). The reduction of silver nitrate to silver nanoparticles by sodium citrate was confirmed by observing a broad absorbance peak between 400–500 nm.

### High resolution-transmission electron microscopy

Further characterization was done using HR-TEM studies. The samples were prepared by placing a drop of the nanoparticle solution onto a carbon-coated copper TEM grid. The samples were then dried under an infrared lamp for a period of 45 min for the solvent to evaporate. High-resolution TEM images were obtained on JEOL TEM model no 2100 instrument operated at an accelerating voltage of 200 kV and 0.23 nm resolution.

### Plant collection and extraction

*Pyrenacantha grandiflora* tubers were collected and extracted using the method provided by Samie and colleagues^7^. The bioactive compounds of *P. grandiflora* were extracted using methanol, hot water (distilled water) and acetone as solvents. All mixtures were filtered through Whatman filter papers and proper actions were taken to ensure that potential active constituents are not lost, distorted or destroyed during the preparation of the extracts from plant samples. Filtrates were concentrated using rotary evaporators (Rota vapor-R, Buchi, Switzerland). Different temperatures were used to evaporate extract solvents; acetone at 50 °C, methanol at 60 °C and chloroform at 59 °C. Hot water extract was concentrated using freeze dryer. All concentrated samples were further dried into powder at room temperature.

### Conjugation of antibiotics and plant extracts

Plant extracts were conjugated to the antibiotics as previously described by Tom and colleagues^17^. The antibiotic solutions (10 mg/ml) were filter-sterilized using a 22 µm filter and refrigerated at 4 °C. *Pyrenacantha grandiflora* tubers compounds were extracted using acetone, water, and methanol as solvents. Each of the plant extracts (10 mg/ml) were mixed with 10 mg/ml of each antibiotic. The mixture was incubated at 30 °C in a rotary shaker for 6 h and then stored at 4 °C for further analysis.

## Conjugation of silver nanoparticles with antibiotics

Antibiotics were prepared by dissolving the respective antibiotic (penicillin, vancomycin, and ampicillin) in distilled water to the concentration of 10 mg/ml. Conjugates were prepared according to Brown and colleagues^3^. The antibiotic solutions were filter-sterilized using 22 µm filter and refrigerated at 4 °C. The combined reducing property of tri-sodium citrate and 10 mg/ml of antibiotics were used to reduce silver nitrate (AgNO3) in order to prepare the conjugates^18^. The conjugates were then stored at 4 °C for further analysis.

### Conjugation of antibiotics, plant extracts, and silver nanoparticles

Antibiotic-silver nanoparticle-plant extract conjugations were done by mixing 10 mg/ml of respective antibiotics (Penicillin, Vancomycin, and Ampicillin), 10 mg/ml of plant extract (methanol, acetone and water extract) and 1 mM Silver nitrate (Sigma, USA) to make 50 ml solution. The mixtures were incubated at 30 °C for 30 min. The conjugates were cooled and stored at 4 °C for further analysis. The formation of the conjugates was denoted by the formation of intense brown colour from colourless. The bottle carrying the mixture was covered with a foil and stored in the dark to avoid the photo-activation of silver nitrate under static conditions.

### Characterization of conjugated antibiotics

All conjugates were analysed using Fourier- transmission electron microscopy (FTIR) in a range of 400–4,000 cm^-1^ to detect various functional groups formed after conjugation that may be responsible for biological activities. Five hundred µl of conjugates were placed on the sample chamber of FTIR spectrophotometer and the spectra were recorded in the scan range of 400–4,000 cm^-1^ with a resolution of 4 cm^-1^ on a Nicolet Avatar 330 FTIR spectrometer.

### Determination of antimicrobial activity of the conjugants

#### Well diffusion assay

The antibacterial activities of the conjugates were determined by well diffusion method as previously described^7^. Clinical and Laboratory Standard Institute (CLSI) guidelines were followed for antimicrobial evaluation. The zones of inhibition were recorded in millimetre (mm). Briefly, bacterial suspensions of *E. coli*, *K. pneumonia, and S. aureus* were prepared with the turbidity of 0.5 McFarland. Mueller–Hinton agar plates were inoculated with that bacterial suspension*.* Wells with a diameter of 6 mm were cut using a cork borer and filled with 30 μl of the conjugated antibiotics and reference samples that were non-conjugated plant extracts, antibiotics and silver nanoparticles. Distilled water was used as a negative control and gentamycin was used as a positive control. All experiments were done in duplicate. Plates were incubated for 24 h at 37 °C. After incubation, the growth inhibition zone diameters were measured and recorded.

#### Microdilution assay

The methods previously described by Samie and colleagues was used to determine the minimum inhibitory concentration following CLSI guidelines for antimicrobial evaluation. The minimum concentration of the antibiotics plus conjugate samples that inhibit the growth of the microorganism are denoted as minimum inhibitory concentration (MIC)^19^. Distilled water was used as negative control and ciprofloxacin was used as positive control. All experiments were done in duplicate. After adding INT (iodo-nitro tetrazolium), the results were read by observing the colour change and determining the MIC. Actively growing microorganisms have the ability to reduce INT to a purple-red colour. Therefore, viable cells turn purple and the dead remains colourless indicating no growth. All samples that showed activity (reduced or no colour change) were inoculated again in the agar plate and incubated overnight to determine the minimum bactericidal concentration (MBC). All tests were done in duplicates.

### Determination of fractional inhibition concentration index (FICI)

Determination of the mutual influence of silver nanoparticles and plant extracts and antibiotics was done using Fractional Inhibition Concentration Index (FICI) as described by Odds and colleagues^20^ using the following formula:

$$FICI=\frac{MIC\,of\,AB}{MIC\,of\,A}+\frac{MIC\,of\,AB}{MIC\,of\,B}$$

where AB represents a combination of *P. grandiflora* tubers extracts (A) and antibiotic (B). Results were interpreted as synergy (FICI ≤ 0.5), antagonism (FICI > 4) and no interaction or additive (FICI > 0.5–4.0).

## Results

### Analysis of silver nanoparticles

**Figure 1:**
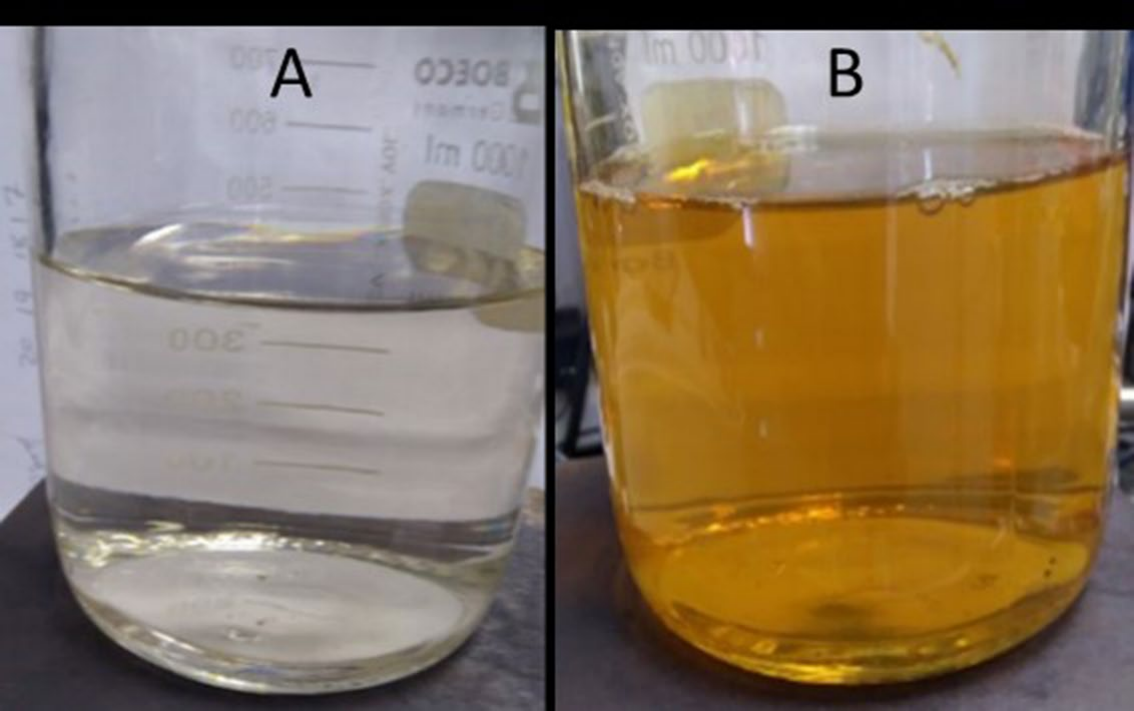
Images illustrating the color change of silver nitrate from colorless (**A**) to yellow (**B**) when citrate was added to confirm the synthesis of silver nanoparticles.

Silver nanoparticles were successfully synthesized using the chemical method. The colour of silver nitrate solution changed from colourless (Fig. 1A) to yellow colour (Fig. 1B) when 1% sodium citrate was added. This colour originates from coherent electron motion in the colloidal solution, giving rise to a characteristic absorption of light at a wavelength of 400–500 nm to confirm the synthesis of nanoparticles.

### UV–Visible analysis of silver nanoparticles

**Figure 2:**
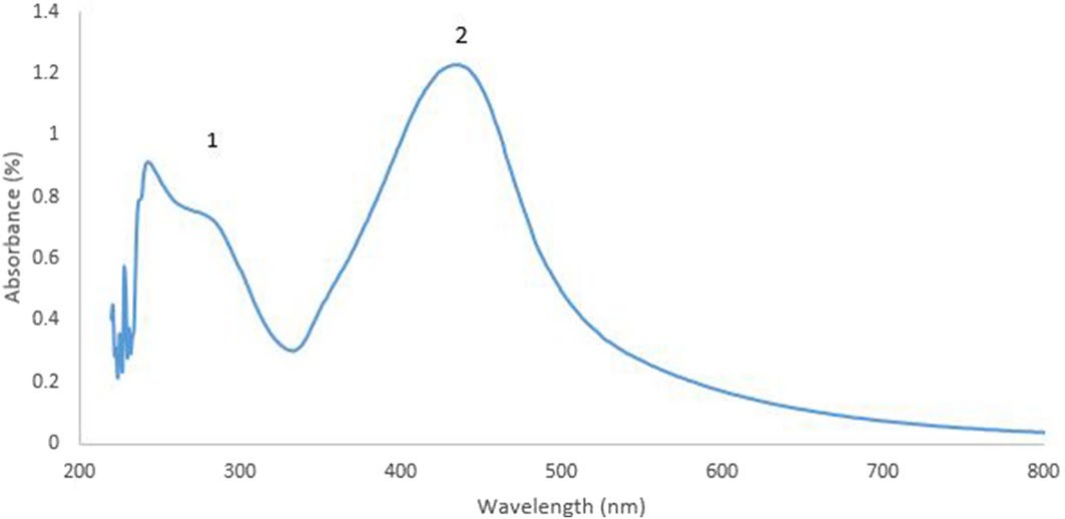
UV–Visible spectra of silver nanoparticles. Peak 1 represents the unreacted silver nitrate and peak 2 represents silver nanoparticles.

Ultra-violet visible spectroscopic studies showed the peak at 434 nm (maximum absorbance) which confirms the presence of silver nanoparticles as shown in peak 2 (Fig. 2). Absorption peak 1, obtained at 290 nm indicates the un-reacted silver nitrate in solution.

### Transmission electron microscopy analysis of silver nanoparticles

**Figure 3:**
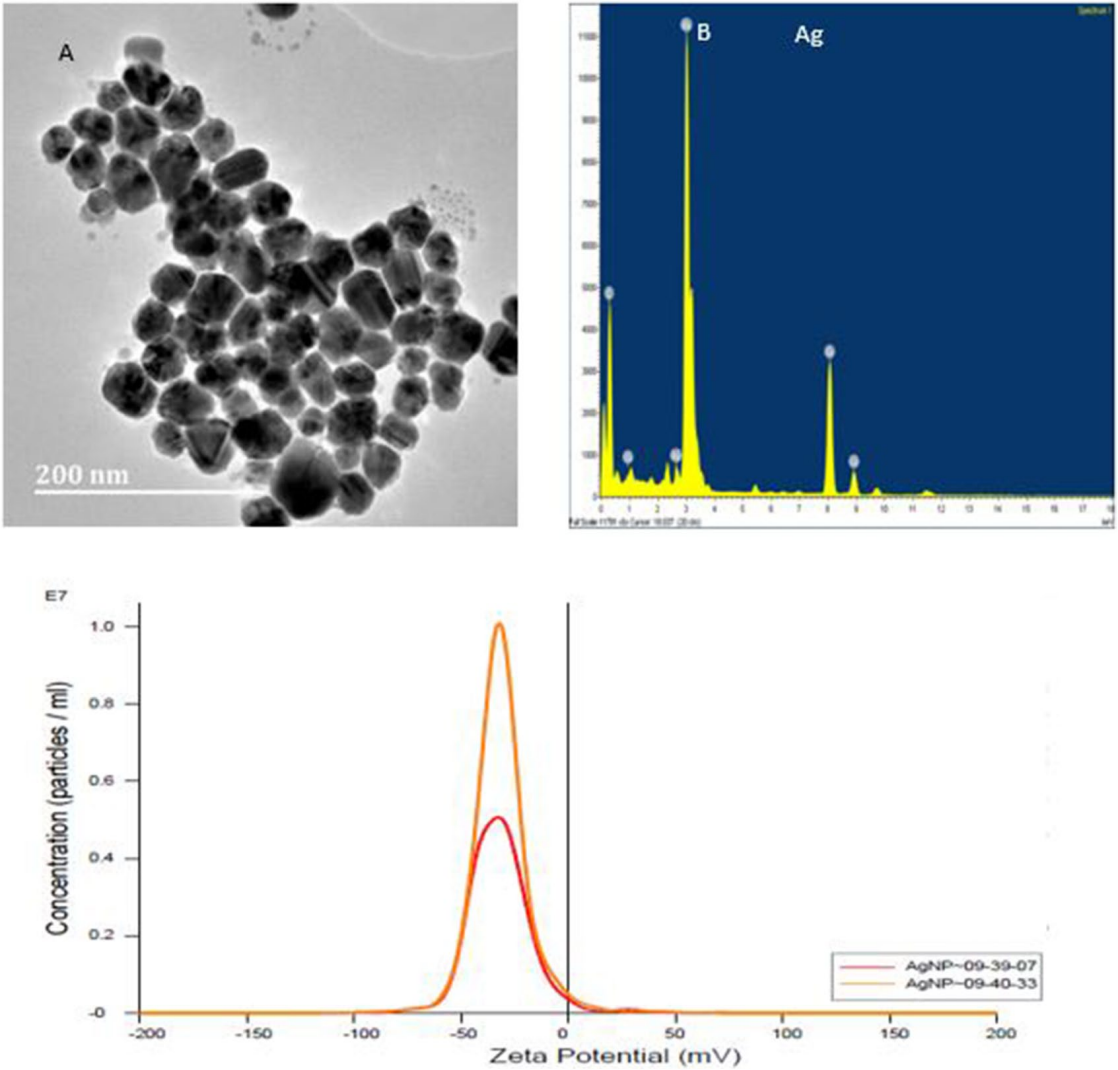
Characteristics of the silver nanoparticles prepared. Images showing the TEM picture of silver nanoparticles (**A**) and EDX pattern (**B**) and the Zeta potential of the AgNPs.

The morphology of nanoparticles was shown to be nanocube shape. In the higher magnification image, silver nanoparticles of 5–33 nm size were also observed (Fig. 3A). However, the average size of the nanoparticles was found to be 13 nm. The EDX analysis confirmed that the particles were composed by elemental silver (Fig. 3B). This results were confirmed using Nanoparticles Tracking Analysis (NTA).

The zeta potential of silver nanoparticles was – 31.5 ± 0.2 mV was obtained from NTA. Zeta potential is used to predict colloidal stability of the solution. A zeta potential of more than 31 mV indicates moderate stability, 41–60 mV good stability, 31–40 mV moderate stability, 10–30 mV incipient instability and 0–5 mV indicates flocculation or coagulation. Particles size distribution was measured as D10: 8.1 + /- 1.8 nm, D50: 13.8 + /- 6.5 nm and D90: 26.8 + /- 11.5 nm. The D10 means that 10% of synthesized silver nanoparticles had the particle size of 8.1 ± 1.8 nm or smaller, similarly to D50 and D90 ^21^.

### FTIR analysis of ampicillin conjugated plant extracts

**Figure 4:**
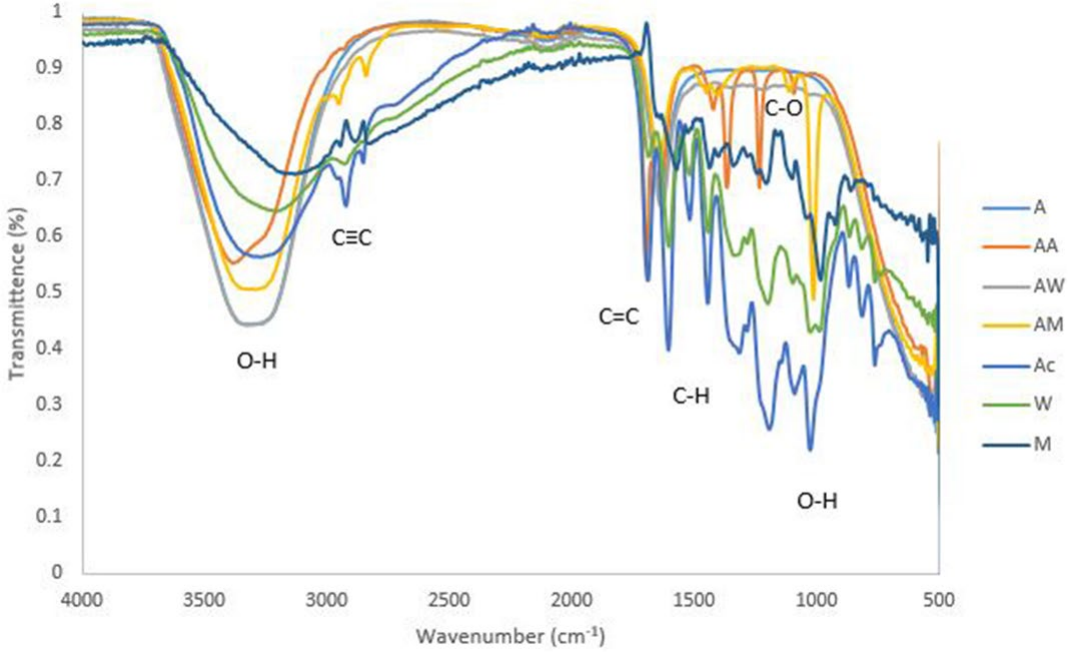
FTIR spectrum of ampicillin conjugates with acetone, water, methanol extracts of *P. grandiflora* as a reference sample, where *A* ampicillin, *AA* ampicillin-acetone extract, *AW* ampicillin-water extract, *AM* ampicillin-methanol extract, *Ac* acetone extract, *M* methanol extract and *W* water extract.

The ampicillin-acetone extracts conjugate exhibited characteristic bands at 3,368 (O–H Stretching), 2068 (C≡C Stretching), 1697 (C = C isolated), 1638 (C = C conjugated), 1,383 (C–H bending), 1,368 (C–H bending), 1,234 (C–O Stretching) and 1,091 (O–H bending) as illustrated in Fig. 1. Bands at 1,383 (C–H bending) and 1,234 (C–O Stretching) were not observed in unconjugated ampicillin nor acetone (Fig. 4) While bands at 2,953 (C–H stretching) and 1,111 (C–O Stretching) were not observed in unconjugated ampicillin nor methanol extracts.

### FTIR analysis of penicillin conjugated plant extracts

**Figure 5:**
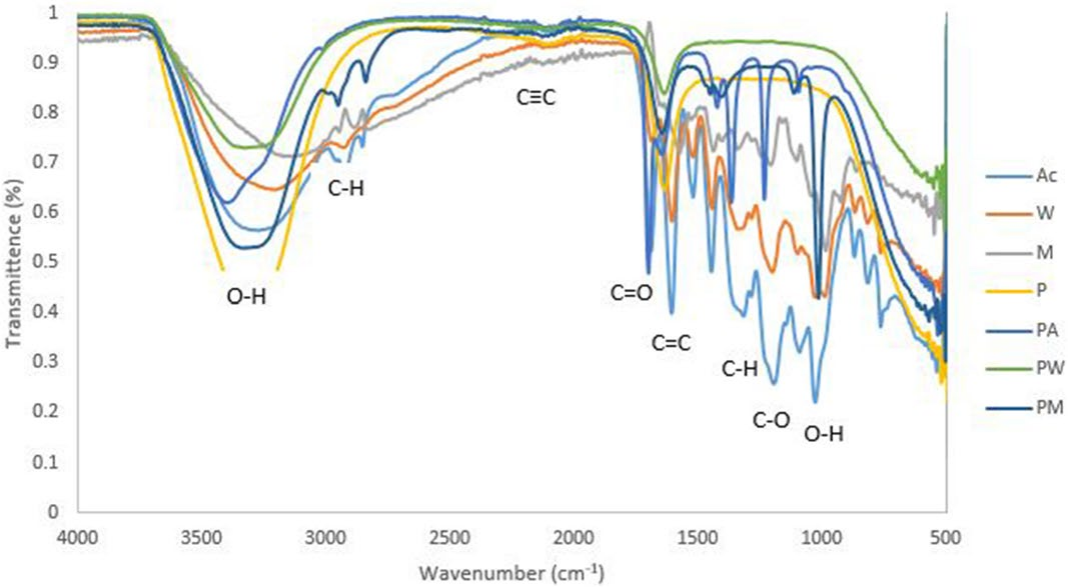
FTIR spectrum of penicillin conjugated with acetone, water and methanol extracts of *P*. *grandiflora* where, *P* penicillin, *PM* penicillin-methanol extract, *PA* penicillin-acetone extract, *PW* penicillin-water extract, *Ac* acetone extract, *M *methanol extract and *W* water extract.

The FTIR spectrum of penicillin and plant extracts conjugates (Fig. 5) illustrated absorption bands at 3,388 (O–H Stretching), 3,272 (C–H Stretching), 3,004 (C–H Stretching), 2,101 (C≡C Stretching), 1701 (C = O group), 1644 (C = C isolated), 1,419 (C–H stretching), 1,360 (C–H bending), 1,232 (C–O Stretching), 1,093 (O–H bending). Only 1,232 (C–O Stretching) and 1,419 (C–H stretching) functional groups were formed in penicillin-acetone extracts whereas in penicillin methanol extracts 2,988 and 2,839 (C–H stretching) were observed as new functional groups.

### FTIR analysis of vancomycin conjugated plant extracts

**Figure 6:**
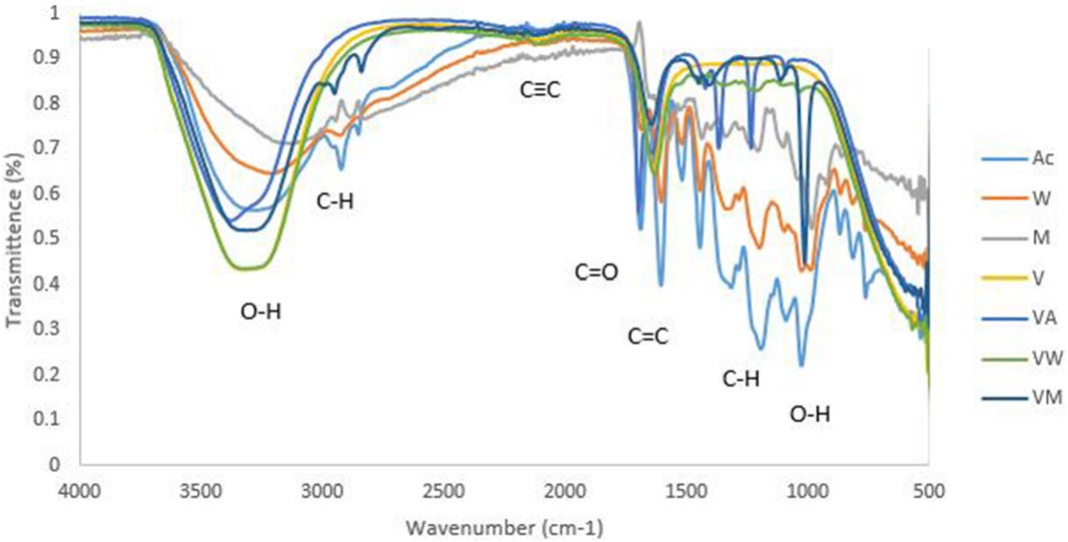
FTIR spectrum of vancomycin conjugated with acetone, water extract and methanol extracts of *P. grandiflora* where, *V* vancomycin, *VA* vancomycin-acetone extract, *VW *vancomycin-water extract, *VM* vancomycin-methanol extract, *A* acetone extract, *M* methanol extract and *W* water extract.

Vancomycin-plant extracts conjugates exhibited characteristic bands at 3,363 (O–H Stretching), 3,255 (C–H Stretching), 2,101 (CC Stretching), 1691 (C = C isolated), 1644 (C = C isolated), 1,419 (C–H Stretching), 3,662 (C–H bending), 1,234 (C–O Stretching) and 1,093 (O–H bending). 3,255 and 1,491(C–H stretching) and1234 (C–O stretching) were the new functional groups formed in vancomycin-acetone extract conjugates (Fig. 6). Hence 2,831 (C–H Stretching), 1,401 (C = C isolated) and 1,009 (C–O Stretching) bands were observed in vancomycin-methanol extracts, but not present in unconjugated vancomycin nor water extracts.

### Antibiotics and silver nanoparticles

**Figure 7:**
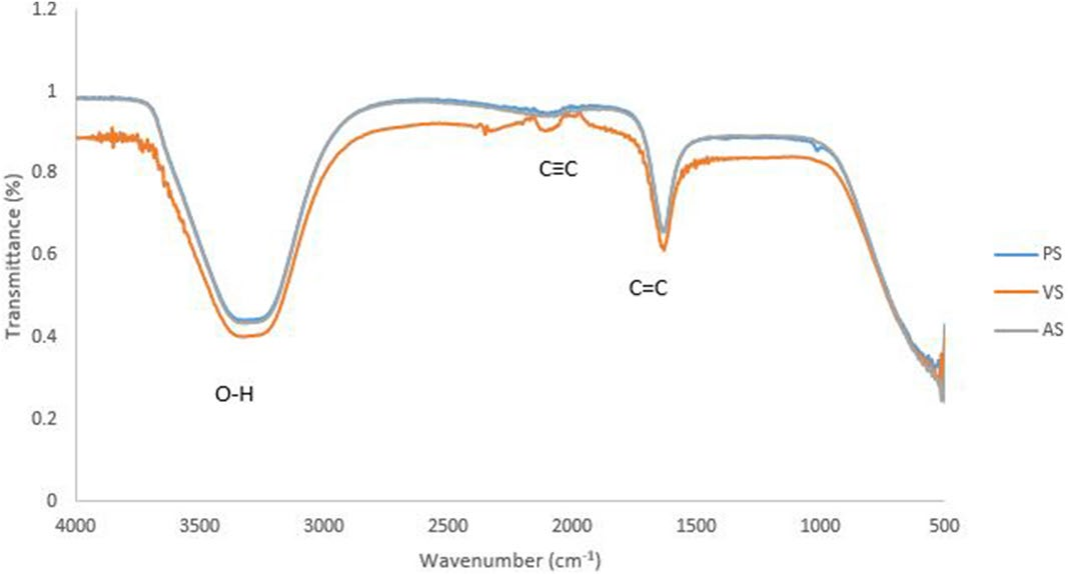
FTIR spectrum of antibiotics conjugated silver nanoparticles where: PS (penicillin conjugated silver nanoparticles); VS (vancomycin conjugated silver nanoparticles) and AS (ampicillin conjugated silver nanoparticles).

From the FTIR spectrum, percent transmission was plotted against wavenumber and the greater the amount of light absorbed by the sample, the smaller the percent transmittance. Different functional groups corresponding to absorbance peaks in the range of 3,000–3,360 (O–H stretching), 2,800–3,000 (C≡C stretching) and 1603–1697 (C = C conjugated) cm^−1^ were observed in almost all antibiotics conjugated silver nanoparticles. However, the difference was observed in the absorbance and peaks at different wavenumbers on same functional groups (Fig. 7). No new functional group was observed when silver nanoparticles were conjugated with penicillin and ampicillin. Vancomycin showed two new C≡C functional group. The peaks are known to be associated with stretching vibrations of hydroxyl groups (O–H) in alcohols or phenolic compounds, CH2 and CH3 functional groups; C = C groups of aromatic compounds or C = O groups of carboxylic acids.

### Conjugation of antibiotics with plant extract and nanoparticles

#### Analysis of ampicillin conjugated silver nanoparticle and plant extracts

**Figure 8:**
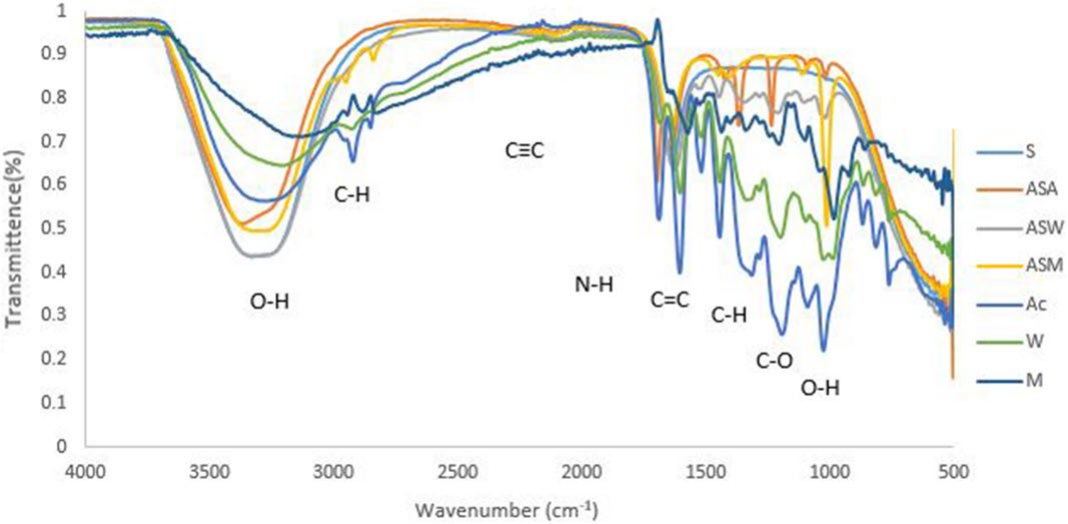
FTIR spectra of ampicillin conjugated silver nanoparticles and plant extracts where: *S* silver nanoparticles, *ASA* ampicillin-silver nanoparticles- acetone extract, *ASW* ampicillin-silver nanoparticles-water extract, *ASM* ampicillin-silver nanoparticles—methanol extract, *A* acetone extract, *M* methanol extract and *W* water extract.

When ampicillin was conjugated with silver nanoparticles and plant extracts it exhibited characteristic bands at 3,350 (O–H Stretching), 3,247 (C = C isolated), 2028 (CC Stretching), 1697 (N–H Stretching), 1644 (C = C isolated), 1,419 (C–H Stretching), 1,362 (C–H bending), 1,234 (C–O Stretching), 1,095 (O–H bending) and 1,020 (C–O Bending) (Fig. 8). Newly formed functional groups from the conjugation included 1,234 (O–H str) and 1,095 (Bending) from ampicillin-silver nanoparticles-acetone extracts (ASA), 2,839 (C–H str) and 1,111(C–O str) from ampicillin-silver nanoparticles-methanol extracts and 1,113 (C=O Str), 1,103 (C–O Str) and 1,020 (O–H bending) from ampicillin-silver nanoparticles-water extracts.

### Analysis of penicillin conjugated silver nanoparticle and plant extracts

**Figure 9:**
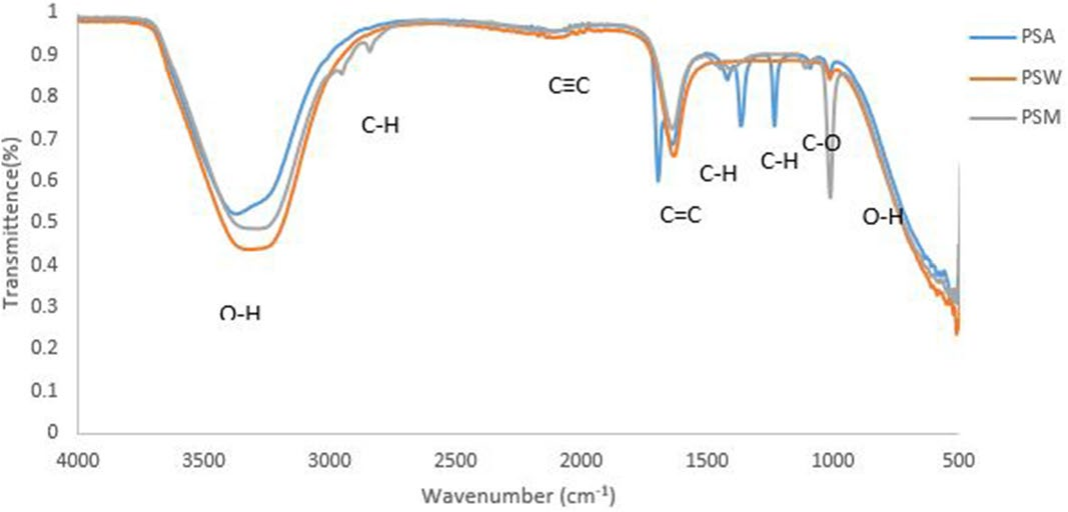
FTIR spectrum of penicillin conjugated silver nanoparticles and plant extracts where, *PSA* penicillin-silver nanoparticles-acetone extract, *PSW* penicillin-silver nanoparticles-water extract, *PSM* penicillin-silver nanoparticles- methanol extract, *S* silver nanoparticles, *A* acetone extract, *M* methanol extract and *W* water extract.

Penicillin conjugated silver nanoparticles and plant extracts exhibited characteristic bands at 3,370 (O–H Stretching), 2082 (C≡C Stretching), 1699 (C = C isolated), 1644 (C = C isolated), 1,423 (C–H bending), 1,368 (C–H Bending), 1,234 (C–O Stretching), 1,095 (O–H bending) and 1,020 (C–O Bending) (Fig. 9). Newly formed functional groups from conjugation included 1,234 (C–O Stretching) and 1,423 (C–H bending) from penicillin conjugated silver nanoparticles and acetone extract, 2,839 and 1,409 (C–H stretching) from penicillin conjugated silver nanoparticles and methanol extract and no new functional group from penicillin conjugated silver nanoparticles and water extracts.

### Analysis of vancomycin conjugated silver nanoparticles and plant extracts

**Figure 10:**
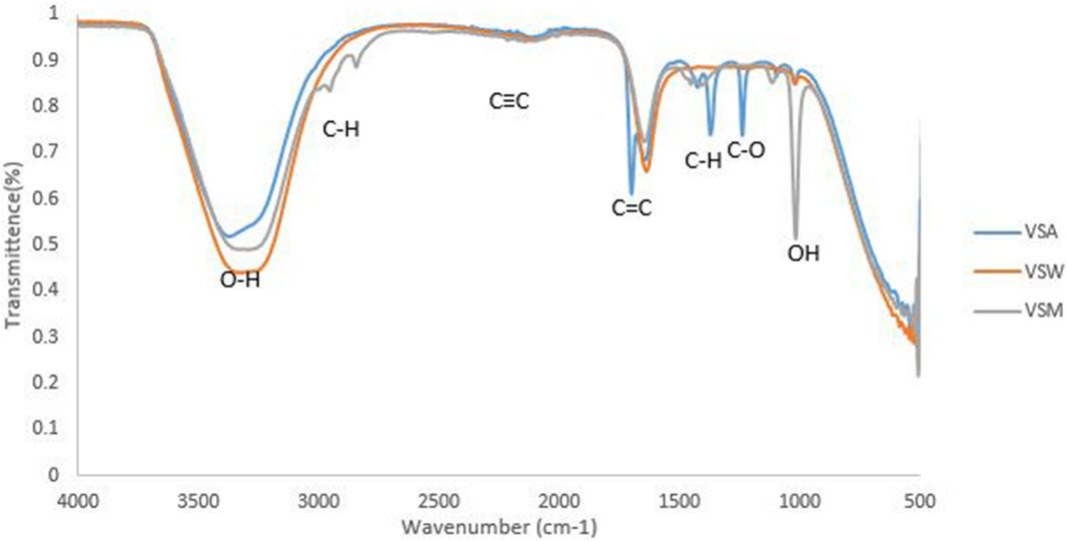
FTIR spectrum of vancomycin conjugated silver nanoparticles and plant extracts where, *VSA* vancomycin-silver nanoparticles- acetone extract, *VSW* Vancomycin-silver nanoparticles-water extract, *VSM* Vancomycin-silver nanoparticles- Methanol extract, *S* silver nanoparticles, *A* acetone extract, *M* methanol extract and *W* water extract.

Vancomycin conjugated silver nanoparticles and plant extracts exhibited characteristic bands at 3,372 (O–H Stretching), 2082 (C≡C Stretching), 1697 (C = C isolated), 1644 (C = C isolated) and 1,011 (C–O Stretching) which were also observed in unconjugated vancomycin, plant extract or silver nanoparticles (Fig. 10). Newly formed functional groups included 1,383 (C–H Bending), 1,368 (C–H Stretching), 1,238 (C–O Stretching) and 1,095 (O–H Bending) from vancomycin conjugated silver nanoparticles and acetone extracts, 2,841, 1,454 and 1,401 (C–H Stretching) and 1,113 (C–O Stretching) from vancomycin conjugated silver nanoparticles and methanol extracts and no new functional group was observed with vancomycin conjugated silver nanoparticles and water extracts.

### Well diffusion assay

#### Antibacterial activity of ampicillin conjugated silver nanoparticle and plant extracts

**Figure 11:**
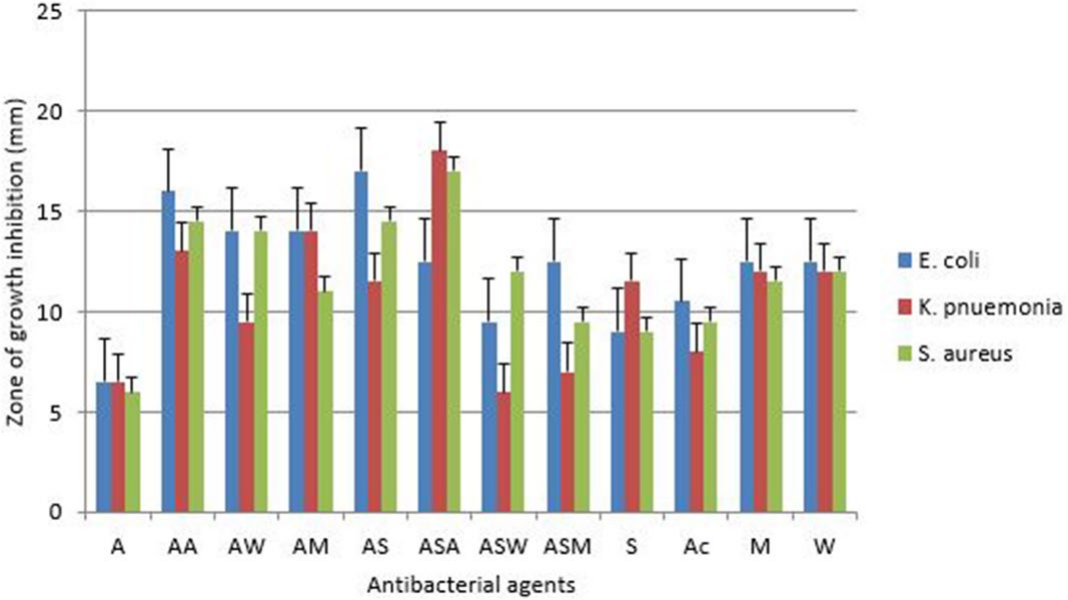
Antibacterial activity of ampicillin conjugated silver nanoparticles and plant extracts against three bacterial where: *A* ampicillin, *AA* ampicillin-acetone extract, *AW* ampicillin-water extract, *AM* ampicillin-methanol extract, *AS* ampicillin-silver nanoparticles, *ASA* ampicillin-silver nanoparticles-acetone extract, *SW* ampicillin-silver nanoparticles-water extract, *ASM *ampicillin-silver nanoparticles-methanol extract, *S* silver nanoparticles, *A* acetone extract, *M* methanol extract and *W* water extract.

Ampicillin was conjugated with silver nanoparticles and plant extracts and their antibacterial activity was evaluated by the well diffusion assay. Conjugated ampicillin was active against all tested bacteria. Antibacterial activity was observed when ampicillin conjugated to the silver nanoparticles and acetone extracts (ASA) against *K. pneumonia* with the highest zone of growth inhibition of 18 mm (Fig. 11). However, very low antibacterial activity was observed when ampicillin was conjugated with silver nanoparticle and water extract against *K. pneumonia* with the zone of growth inhibition of 6 mm.

#### Antibacterial activity of penicillin conjugated silver nanoparticles and plants extracts

**Figure 12:**
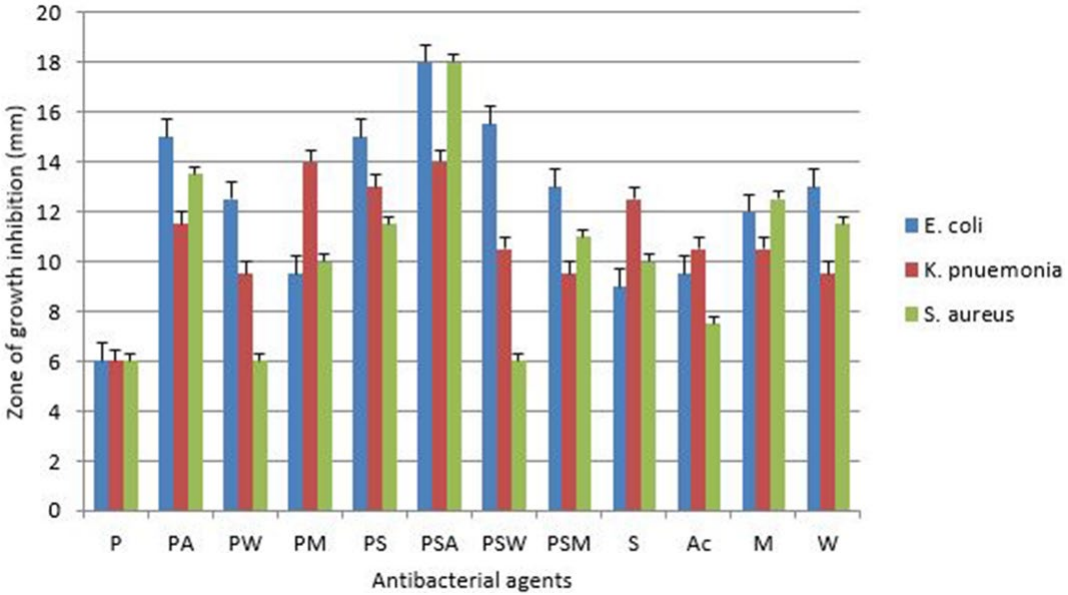
Antibacterial activity of penicillin-conjugated silver nanoparticles and plant extracts where, *P* penicillin, *PM* penicillin-methanol extract, *PA* penicillin-acetone extract, *PW* penicillin-water extract, *PS* penicillin-silver nanoparticles, *PSA* penicillin-silver nanoparticles-acetone extract, *PSW* penicillin-silver nanoparticles-water extract, *PSM* penicillin-silver nanoparticles-methanol extract, *S* silver nanoparticles, *A* acetone extract, *M* methanol extract and *W* water extract.

Antibacterial activity was studied against three pathogenic resistant bacterial strains with well diffusion assay. Interesting results were observed with silver nanoparticles conjugated with antibiotic and plant extract especially the acetone extract. The antibacterial activity of conjugated penicillin is shown in Fig. 12. The activity of penicillin shows a massive increase when conjugated with acetone extract and silver nanoparticle against *E. coli* and *S. aureus* with the highest zone of growth inhibition of 18 mm. Most conjugated penicillin was most active against *E. coli* while MRSA showed resistance to penicillin-water extract and to penicillin-water extract-silver nanoparticles.

#### Antibacterial activity of vancomycin conjugated silver nanoparticles and plants extract

**Figure 13:**
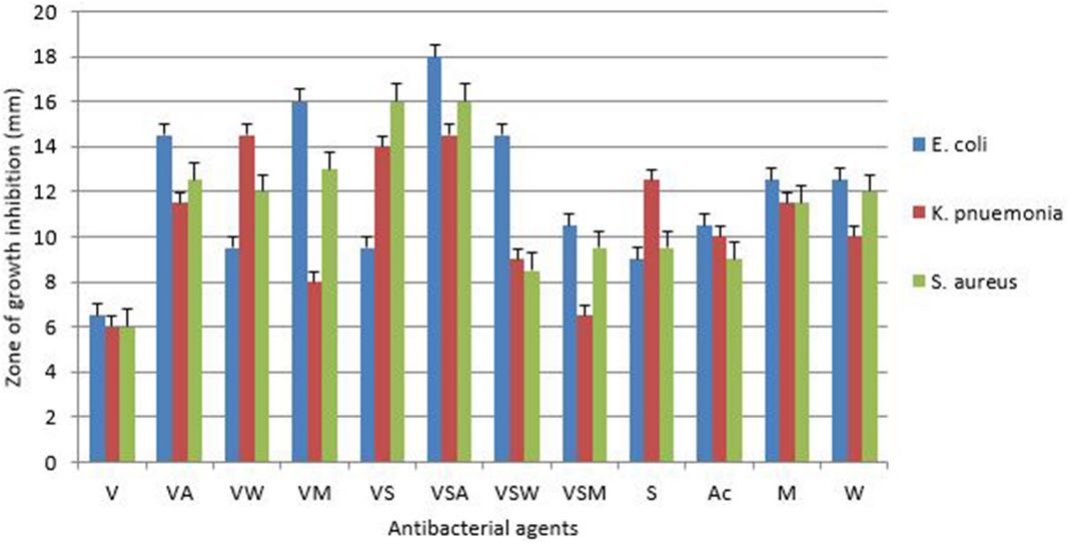
Antibacterial activity of vancomycin conjugated silver nanoparticles and plant extracts on three bacterial strains, Where: *V* vancomycin, *VA* vancomycin-acetone extract, *VW* vancomycin-water extract, *VM* vancomycin-methanol extract, *VS* vancomycin-silver nanoparticles, *VSA* vancomycin-silver nanoparticles-acetone extract, *VSW* vancomycin-silver nanoparticles-water extract, *VSM* vancomycin-silver nanoparticles-methanol extract, *S* silver nanoparticles, *A *acetone extract, *M* methanol extract and *W* water extract.

The highest activity was observed when vancomycin was conjugated with silver nanoparticles and acetone extract against *E. coli* with a zone of growth inhibition of 18 mm (Fig. 13). Vancomycin-silver nanoparticles-water extract and vancomycin-water extract showed a minor increase of antibacterial activity of 9 mm and 8 mm respectively against *K. pneumoniae*.

### Microdilution assay

#### Minimum inhibitory concentration of conjugated ampicillin

Microdilution assay was used to determine the minimum inhibitory concentration of ampicillin conjugated plant extracts and silver nanoparticles. The concentration used range from 0.8 to 0.0063 mg/ml (Table 1). All the tests were conducted in duplicate. Ampicillin conjugated with silver nanoparticles and plant extracts exhibited the lowest MIC value of 0.0063 mg/ml against *K. pneumoniae*. Ampicillin conjugated silver nanoparticles showed the lower MIC value of 0.05 mg/ml against *E. coli* and 0.3 mg/ml against *S. aureus*. However, ampicillin alone needed more than 0.8 mg/ml to inhibit the growth of *E. coli*, *K. pneumoniae* or *S. aureus*.

**Table1:** Minimum inhibitory concentration (MIC) of ampicillin conjugated plant extracts and silver nanoparticles where.

Sample	*E. coli* (mg/ml)	*K. pneumonia* (mg/ml)	*S. aureus* (mg/ml)
A	0.8 ± 0.00	0.8 ± 0.00	0.8 ± 0.00
AA	0.4 ± 0.00	0.2 ± 0.00	0.4 ± 0.00
AW	0.8 ± 0.00	0.3 ± 0.14143	0.2 ± 0.00
AM	0.8 ± 0.00	0.4 ± 0.00	0.075 ± 0.03536
AS	0.05 ± 0.00	0.8 ± 0.00	0.3 ± 0.14143
ASA	0.3 ± 0.14143	0.4 ± 0.00	0.4 ± 0.00
ASW	0.8 ± 0.00	**0.0063 ± 0.00**	0.4 ± 0.00
ASM	0.6 ± 0.28284	0.075 ± 0.03536	0.6 ± 0.28284
S	0.4 ± 0.00	0.3 ± 0.14143	0.3 ± 0.14143
Ac	0.6 ± 0.28284	0.6 ± 0.28284	0.15 ± 0.07071
W	0.8 ± 0.00	0.8 ± 0.00	0.8 ± 0.00
M	0.4 ± 0.00	0.3 ± 0.14143	0.3 ± 0.14143

The results were recorded as mean ± standard deviation.

*A* ampicillin, *AA* ampicillin-acetone extract, *AW* ampicillin-water extract, *AM* ampicillin-methanol extract, *AS* ampicillin-silver nanoparticles, *ASA* ampicillin-silver nanoparticles-acetone extract, *ASW* ampicillin-silver nanoparticles-water extract, *ASM* ampicillin-silver nanoparticles—methanol extract, *S* silver nanoparticles, *Ac* acetone extract, *W* water extract, *M *methanol extract.

#### Minimum inhibitory concentration (MIC) of conjugated penicillin

Minimum inhibitory concentration of conjugated penicillin was determined by microdilution assay. The concentration used ranged from 0.8–0.0063 mg/ml (Table 2). Distilled water was used as a negative control and gentamycin was used as positive control. All the tests were conducted in duplicate. More than 0.8 mg/ml of penicillin was needed to inhibit the growth of *E. coli*, *K. pneumoniae,* and *S. aureus*. Lowest MIC values were observed with penicillin conjugated silver nanoparticles giving an MIC of 0.0125 mg/ml against *S. aureus.* Penicillin conjugated methanol extract and also with silver nanoparticles were effective in inhibiting the growth of *E. coli* at MIC values of 0.075 and 0.025 mg/ml.

**Table 2:** Minimum inhibitory concentration (MIC) of penicillin conjugated plant extracts and silver nanoparticles.

Sample	*E. coli* (mg/ml)	*K. pneumonia* (mg/ml)	*S. aureus* (mg/ml)
P	0.8 ± 0.00	0.8 ± 0.00	0.8 ± 0.00
PA	0.3 ± 0.14143	0.15	0.4 ± 0.00
PW	0.4 ± 0.00	0.4 ± 0.00	0.4 ± 0.00
PM	0.075 ± 0.03536	0.6 ± 0.28284	0.3 ± 0.14143
PS	0.075 ± 0.03536	0.6 ± 0.28284	0.0,125 ± 0.00
PSA	0.6 ± 0.28284	0.6 ± 0.28284	0.6 ± 0.28284
PSW	0.8 ± 0.00	0.025 ± 0.00	0.8 ± 0.00
PSM	0.025 ± 0.00	0.8 ± 0.00	0.8 ± 0.00
S	0.4 ± 0.00	0.3 ± 0.14143	0.3 ± 0.14143
Ac	0.6 ± 0.28284	0.6 ± 0.28284	0.15 ± 0.07071
W	0.8 ± 0.00	0.8 ± 0.00	0.8 ± 0.00
M	0.4 ± 0.00	0.3 ± 0.14143	0.3 ± 0.14143

The results were recorded as mean ± standard deviation.

*P* penicillin, *PM* penicillin-methanol extract, *PA* penicillin-acetone extract, *PW* penicillin-water extract, *PS* penicillin-silver nanoparticles, *PSA* penicillin-silver nanoparticles-acetone extract, *PSW* penicillin-silver nanoparticles-water extract, *PSM* penicillin-silver nanoparticles-methanol extract, *S* silver nanoparticles, *Ac* acetone extract, *W* water extract, *M* methanol extract.

#### Minimum inhibition concentration of conjugated vancomycin

Minimum inhibitory concentrations of conjugated vancomycin were tested against three pathogenic bacteria using microdilution assay. All the tests were conducted in duplicate. The MIC values obtained from vancomycin conjugated plant extracts and silver nanoparticles are shown in Table 3. The MIC of conjugated vancomycin was seen in the range of 0.8–0.0063 mg/ml (Table 3). The lowest concentration to inhibit the growth of *E. coli* amongst the conjugate was observed when vancomycin is conjugated with silver nanoparticles and methanol extracts with MIC value of 0.0125 mg/ml.

**Table 3:** Minimum inhibitory concentration (MIC) of vancomycin conjugated plant extracts and silver nanoparticles.

Sample	*E. coli* (mg/ml)	*K. pneumonia* (mg/ml)	*S. aureus* (mg/ml)
V	0.8 ± 0.00	0.6 ± 0.2828	0.8 ± 0.00
VA	0.8 ± 0.00	0.4 ± 0.00	0.3 ± 0.14143
VW	0.6 ± 0.28284	0.4 ± 0.00	0.225 ± 0.24749
VM	0.8 ± 0.00	0.3 ± 0.14143	0.6 ± 0.2828
VS	0.15 ± 0.07071	0.05 ± 0.00	0.1 ± 0.00
VSA	0.4 ± 0.00	0.4 ± 0.00	0.4 ± 0.00
VSW	0.8 ± 0.00	0.6 ± 0.2828	0.6 ± 0.2828
VSM	0.0125 ± 0.00	0.4 ± 0.00	0.4 ± 0.00
S	0.4 ± 0.00	0.3 ± 0.14143	0.3 ± 0.14143
Ac	0.6 ± 09 .2828	0.6 ± 0.2828	0.15 ± 0.07071
W	0.8 ± 0.00	0.8 ± 0.00	0.8 ± 0.00
M	0.4 ± 0.00	0.3 ± 0.14143	0.3 ± 0.14143

The results were recorded as mean ± standard deviation.

*V* vancomycin, *VA* vancomycin-acetone extract, *VW* vancomycin-water extract, *VM* vancomycin-methanol extract, *VS* vancomycin-silver nanoparticles, *VSA* vancomycin-silver nanoparticles-acetone extract, *VSW* vancomycin-silver nanoparticles-water extract, *VSM* vancomycin-silver nanoparticles-methanol extract, *S* silver nanoparticles, *Ac* acetone extract, *W* water extract, *M* methanol extract.

### Determination of fractional inhibition concetration index (FICI)

#### Fractional inhibition concentration index of conjugated ampicillin

The determination of the mutual influence of antibiotics, silver nanoparticles and plant extracts in the conjugates was done using fractional inhibition concentration index (FICI). Fractional inhibition concentration index was calculated on the results obtained from MIC and the results are recorded in Table 4. A total of seven samples of conjugated ampicillin were tested against three bacterial ATCC strains. Only 3 (14.29%) out of all reactions were synergistic, while 16 (76.19%) were additive and 2 (9.52%) were antagonistic.

**Table 4:** Effect of conjugating ampicillin with plant extracts and silver nanoparticles.

Sample	*E. coli*	*K. pneumoniae*	*S. aureus*
AA	1.1667 (A)	0.5833 (A)	3.1667 (A)
AW	2 (A)	0.75 (A)	0.5 (A)
AM	3 (A)	1.833 (A)	**0.34375 (S)**
AS	**0.1875 (S)**	3.667 (A)	1.375 (A)
ASA	1.625 (A)	2.5 (A)	4.5 (N)
ASW	4 (A)	**0.03675 (S)**	2.3333 (A)
ASM	3.75 (A)	0.59375 (A)	4.75 (N)

Key: A (Additive), N (Antagonism) and S (synergy).

*A* ampicillin, *AA* ampicillin-acetone extract, *AW* ampicillin-water extract, *AM* ampicillin-methanol extract, *AS* ampicillin-silver nanoparticles, *ASA* ampicillin-silver nanoparticles-acetone extract, *ASW* ampicillin-silver nanoparticles-water extract, *ASM* ampicillin-silver nanoparticles-methanol extract.

#### Fractional inhibition concentration index of conjugated penicillin

Fractional inhibition concentration index of penicillin conjugates was calculated from the obtained MIC results of conjugated penicillin and recorded in Table 5. *E. coli, K. pneumonia,* and *S. aureus* were used to determine the mutual effect of penicillin conjugated with plant extracts and nanoparticles. 6 synergies (28.57%) were observed, 11 (52.38%) were additive and 4 (19.05%) were antagonisms.

**Table 5:** Effect of conjugating penicillin with plant extracts and silver nanoparticles.

Sample	*E. coli*	*K. pneumoniae*	*S. aureus*
PA	0.875 (A)	**0.4375 (S)**	3.1667 (A)
PW	1 (A)	1 (A)	1 (A)
PM	**0.2 (S)**	2.75 (A)	1.375 (A)
PS	**0.2 (S)**	2.75 (A)	**0.05729 (S)**
PSA	3.25 (A)	3.75 (A)	6.75 (N)
PSW	4 (A)	**0.145833 (S)**	4.6667 (N)
PSM	**0.15625 (S)**	6.33 (N)	6.3333 (N)

Key: A (Additive), N (Antagonism) and S (synergy).

*P* penicillin-methanol extract, *PA* penicillin-acetone extract, *PW* penicillin-water extract, *PS* penicillin-silver nanoparticles, *PSA* penicillin-silver nanoparticles-acetone extract, *PSW* penicillin-silver nanoparticles- water extract, *PSM* penicillin-silver nanoparticles-methanol extract, *S* silver nanoparticles, *A* acetone extract, *M* methanol extract and *W* = water extract.

#### Fractional inhibition concentration index of conjugated vancomycin

The FICI results of conjugated vancomycin are recorded in Table 6. A total of seven sample were tested against three ATCC bacterial strains. Only 3 synergy (14.29%) was observed, 17(80.95%) were additive and 1 (4.76%) were antagonism.

**Table 6:** Effect of conjugating vancomycin with plant extracts and silver nanoparticles.

Sample	*E. coli*	*K. pneumoniae*	*S. aureus*
VA	2.3333 (A)	1.3333 (A)	2.375 (A)
VW	1.5 (A)	1.16667 (A)	0.5625 (A)
VM	3 (A)	1.5 (A)	2.75 (A)
VS	0.5625 (A)	**0.25 (S)**	**0.458333 (S)**
VSA	2.16667 (A)	2.6667 (A)	4.5 (N)
VSW	**0.078125 (S)**	3.75 (A)	3.5 (A)
VSM	4 (A)	3.3333 (A)	3.16667 (A)

Key: A (Additive), N (Antagonism) and S (synergy).

*V* vancomycin, *VA* vancomycin- acetone extract, *VW *vancomycin-water extract, *VM* vancomycin-methanol extract, *VS* vancomycin-silver nanoparticles, *VSA* vancomycin-silver nanoparticles-acetone extract, *VSW* vancomycin-silver nanoparticles-water extract, *VSM* vancomycin-silver nanoparticles-methanol extract.

## Discussion

The present study has demonstrated antibacterial activity of antibiotics conjugated to plant extracts and silver nanoparticles. Ampicillin, penicillin, and vancomycin are now less effective on their own against pathogenic bacteria^22^. Bio-conjugation allows the formation of chemical bonds between the biological molecules and stabilizer molecules which is attached to the surface of the nanoparticles^23^. Biomolecules that are found in the plant extracts (e.g., flavonoids, phenols, peptides, etc.) are responsible for reducing and stabilizing metal ion during synthesis of nanoparticles. ^15^.

Formation of the new functional groups was identified by FTIR. When ampicillin was conjugated with plant extracts, 1,383 (C–H bending) and 1,234 (C–O Stretching) were observed with acetone extract, and 2,953 (C–H stretching) and 1,111 (C–O Stretching) were observed with methanol extracts. These functional groups were also observed in the ampicillin FTIR spectra when conjugated to nanoparticles, hence this showed that new bonds were formed when plants extract were conjugated to silver nanoparticles^24^.The adsorption or binding of the antibiotic around the surface of the nanoparticles is indicated by the presence of characteristic bands in FTIR spectra, in penicillin methanol extracts 2,988 and 2,839 (C–H stretching) were observed as new functional group while 2,831 (C–H Stretching), 1,401 (C = C isolated) and 1,009 (C–O Stretching) bands where observed in vancomycin-methanol extracts.

From well diffusion assay conjugated ampicillin were effective against all tested bacteria. Though, most effective antibacterial activity was observed when ampicillin was conjugated with silver nanoparticle and acetone extracts (ASA) against *K. pneumonia*. Similarly, another study reported a significant increase in zones of inhibition of silver nanoparticles conjugated with ampicillin as compared to ampicillin alone^25^. Penicillin showed a massive increase of antibacterial activity when conjugated with acetone extract and silver nanoparticle (PSA) against *E. coli* and *S. aureus*, when vancomycin is conjugated with silver nanoparticles and acetone extracts also show high efficacy as antibacterial agents. Hence, these results indicate that *P. grandiflora* tubers acetone extracts and silver nanoparticles in conjugation with selected antibiotics in this study exhibit effective antibacterial activity.

Minimum inhibition concentration in this study ranged from 0.8 to 0.0063 which are comparatively higher values than the results previously described^19^. When ampicillin was conjugated with silver nanoparticles and water extracts (ASW) the conjugant inhibited the growth of *K. pneumonia* giving the lowest MIC value of 0.0063 mg/ml, which is good for the antimicrobial compound in order to avoid toxicity to host cell. Similarly, penicillin conjugated silver nanoparticles (PS) inhibits the growth of *S. aureus* with the lowest MIC value of 0.0125 mg/ml. Polymeric nanoparticles were also reported to display more effective antimicrobial activity against methicillin-resistant *Staphylococcus aureus* when compared with general antibiotics^8,26^. The lowest concentration to inhibit the growth of *E. coli* amongst the conjugates was observed when vancomycin was conjugated with silver nanoparticles and methanol extracts (VSM) with MIC value of 0.0125 mg/ml. This suggests that these nanoparticle conjugates can be used to treat multidrug resistant bacteria according to the study conducted by Kora and colleagues^27^.

At present, there is a great deal of extension significance for the advancement of new antimicrobials in the treatment of diseases caused by microbes. The most recent pattern display that the plant-based antimicrobial agents have a huge therapeutic potential since they do not show any significant side effects on individuals^28^. Mutual influence of antibiotics, silver nanoparticles and plant extracts in conjugates were determined with fractional inhibition concentration index (FICI). Synergistic effects were observed with ampicillin-methanol (AM) and penicillin-silver nanoparticles (PS) against S. *aureus*, penicillin-silver nanoparticle-methanol (PSM) and vancomycin-silver nanoparticles-water (VSW) against *E. coli*. A study on ampicillin conjugated silver nanoparticles functionalized with polyvinyl pyrrolidone showed maximum antibacterial activity compared to other silver nanoparticles conjugates^29^. This study exhibited significant results compared to the antibiotics alone. Improvements in this regard can be achieved with cytotoxicity testing first and then deciding whether an antibiotic will help the patient or not^30^.

## Conclusion

The overall results indicate that conjugated antibiotics with *P. grandiflora* and silver nanoparticles are medicinally important and can be used to improve the activity of existing antibiotics that have become less effective on their own. Further analysis to purify these conjugates are needed.
